# Cold for centuries: a brief history of cryotherapies to improve health, injury and post-exercise recovery

**DOI:** 10.1007/s00421-022-04915-5

**Published:** 2022-02-23

**Authors:** Robert Allan, James Malone, Jill Alexander, Salahuddin Vorajee, Mohammed Ihsan, Warren Gregson, Susan Kwiecien, Chris Mawhinney

**Affiliations:** 1 Centre for Applied Sport Physical Activity and Performance, University of Central Lancashire, Preston, UK; 2 School of Health Sciences, Liverpool Hope University, Liverpool, UK; 3 Human Potential Translational Research Program, National University of Singapore, Singapore, Singapore; 4 Football Exchange, Research Institute of Sports Sciences, Liverpool John Moores University, Liverpool, UK; 5 Nicholas Institute of Sports Medicine and Athletic Trauma, Lenox Hill Hospital, New York, NY USA; 6 College of Sports Science and Technology, Mahidol University, Bangkok, Thailand; 7 Football Performance Hub, Institute of Coaching and Performance (ICaP), School of Sport and Health Sciences, University of Central Lancashire, Preston, UK

**Keywords:** Ice, Cold-water immersion, Phase change material, Cold air, Historical

## Abstract

For centuries, cold temperatures have been used by humans for therapeutic, health and sporting recovery purposes. This application of cold for therapeutic purposes is regularly referred to as cryotherapy. Cryotherapies including ice, cold-water and cold air have been popularised by an ability to remove heat, reduce core and tissue temperatures, and alter blood flow in humans. The resulting downstream effects upon human physiologies providing benefits that include a reduced perception of pain, or analgesia, and an improved sensation of well-being. Ultimately, such benefits have been translated into therapies that may assist in improving post-exercise recovery, with further investigations assessing the role that cryotherapies can play in attenuating the ensuing post-exercise inflammatory response. Whilst considerable progress has been made in our understanding of the mechanistic changes associated with adopting cryotherapies, research focus tends to look towards the future rather than to the past. It has been suggested that this might be due to the notion of progress being defined as change over time from lower to higher states of knowledge. However, a historical perspective, studying a subject in light of its earliest phase and subsequent evolution, could help sharpen one’s vision of the present; helping to generate new research questions as well as look at old questions in new ways. Therefore, the aim of this brief historical perspective is to highlight the origins of the many arms of this popular recovery and treatment technique, whilst further assessing the changing face of cryotherapy. We conclude by discussing what lies ahead in the future for cold-application techniques.

## Introduction

**Fig. 1:**
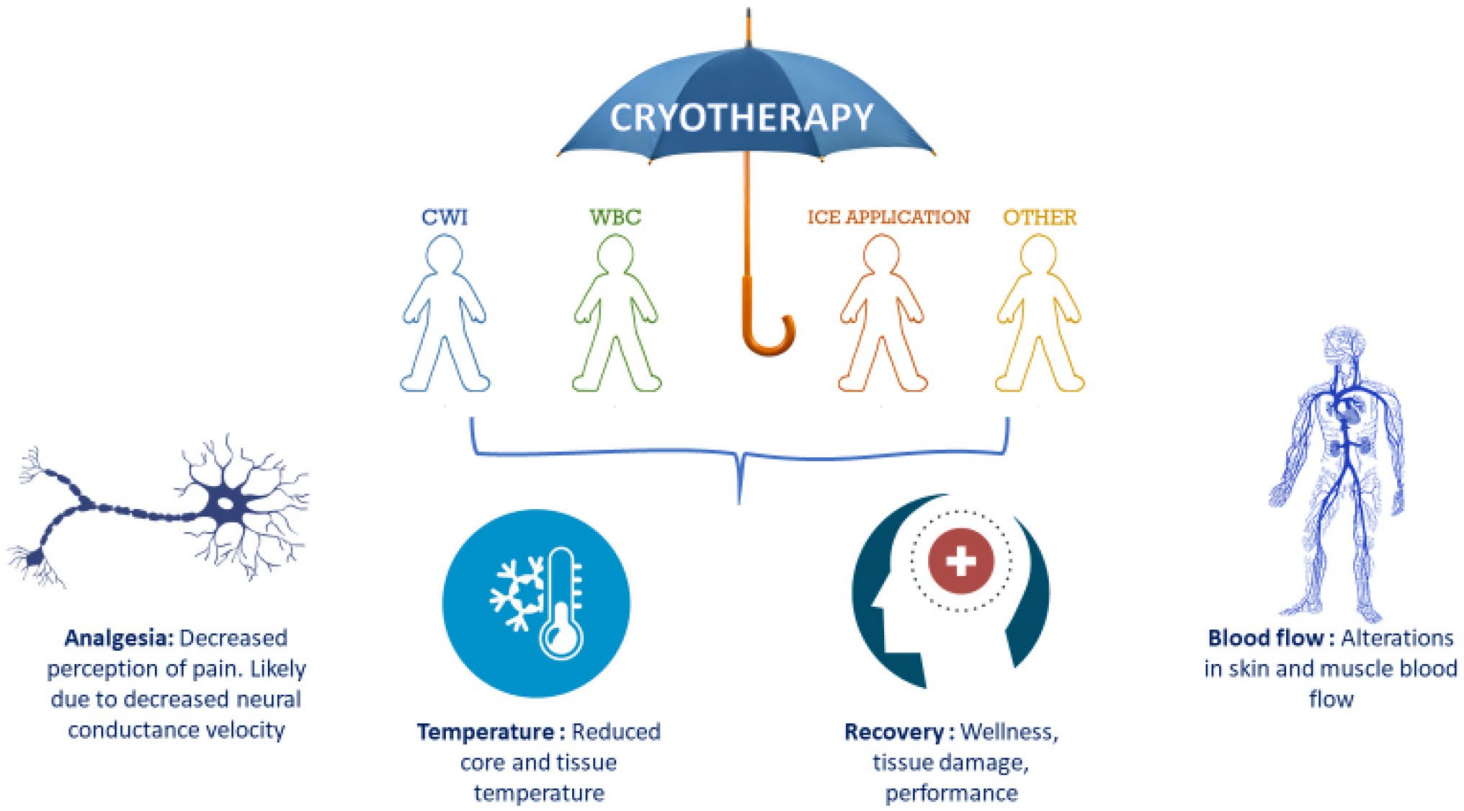
The cryotherapy umbrella and mechanisms of action. CWI; cold-water immersion, WBC; whole-body cryotherapy, other; inclusive of cryo-compression devices, phase change material

**Fig. 2:**
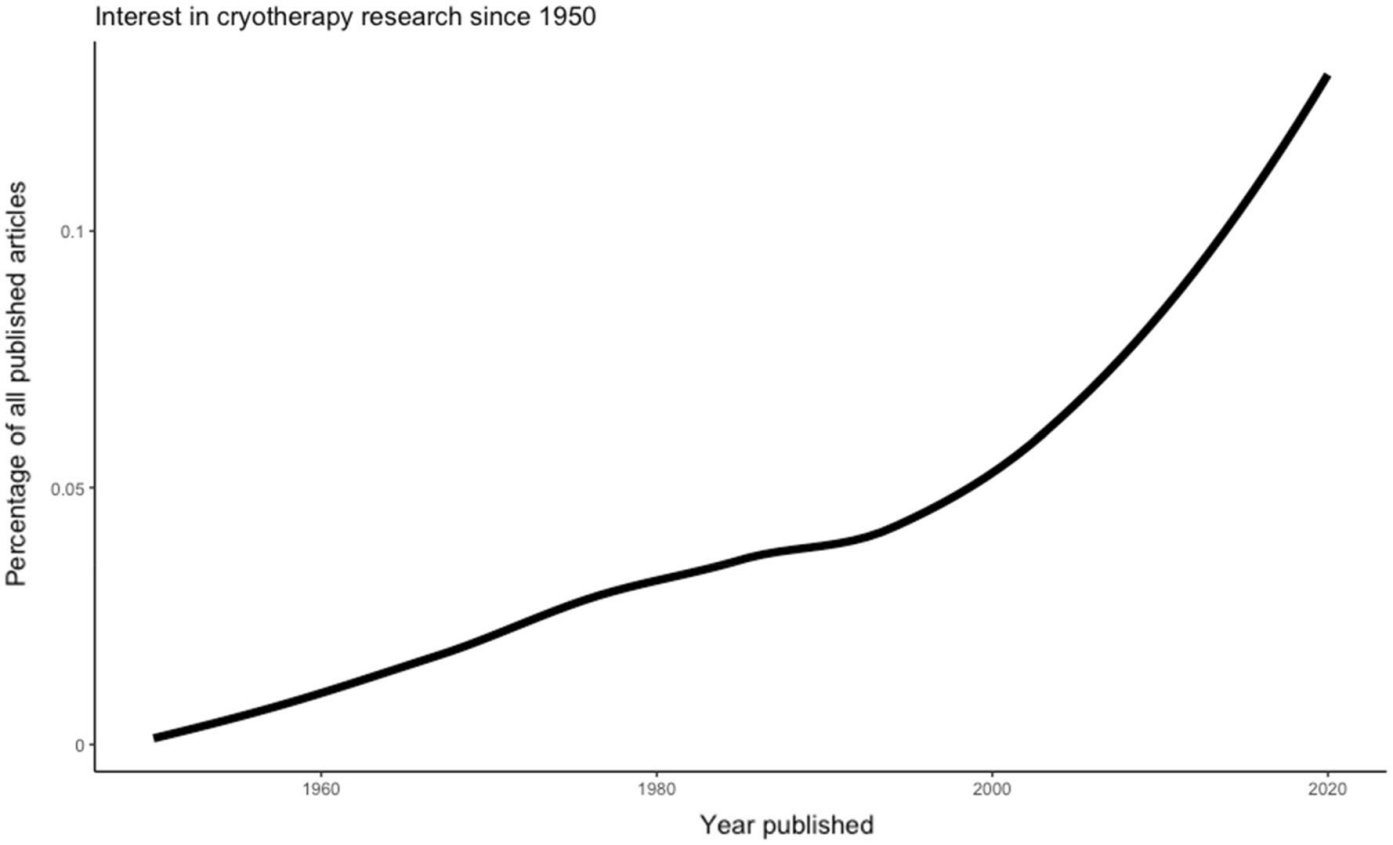
Interest in cryotherapy research since 1950. Data were collected using the “europepmc” R package (Jahn and Salmon 2021) using the search term “cryotherapy” and displayed as a percentage of all publications in the given year. The year-to-year trend is displayed by overlaying a loess smoothed fit curve on the data points

Cryotherapy is an umbrella term often used to describe therapeutic processes involving cold temperatures (Fig. 1). The principle purpose of cryotherapy is the withdrawal of heat (Kwiecien and McHugh 2021) assisted by way of reductions in core and tissue temperatures (Stephens et al. 2018; Vromans et al. 2019) and alterations in blood flow (Mawhinney et al. 2017a, b, 2020). Physiologically, the efficacy of cryotherapy is seen to be important primarily due to analgesic benefits (Murray and Cardinale 2015) associated with slowing sensory nerve conduction velocity (Herrera et al. 2010; Algafly and George 2007; Ernst and Fialka 1994). Historically, this has led to cryotherapy gaining much attention (Fig. 2), with its application long been thought as efficient in the treatment of primary and secondary tissue damage and the ensuing inflammatory response. However, in-depth discussion of the underpinning physiology of cryotherapy is beyond the scope of the current article; as such, readers are directed to recent reviews highlighted by ourselves and others (Kwiecien and McHugh 2021; Peake et al. 2017).

Over time, the way in which cold temperature has been applied to the human body has remained largely unchanged, with the application of ice, cold-air and cold-water maintaining popularity. The evolution of practice seemingly focussed on the cold dose applied through strict control of the temperature and duration of the cooling stimulus. Many aspects of historical physiology can be traced as far back as ancient Greece (West 2014). Indeed, the use of cryotherapy for benefits to health, treatment and recovery dates back centuries. Work by Hippocrates suggested water therapy could ‘allay lassitude’ in reducing the depletion of energy or strength (Tipton et al. 2017); the mention of ice and snow in relation to oedema leading to some crediting him as the grandfather of cryotherapy (Kwiecien and McHugh 2021; Rivenburgh 1992). Whilst considerable progress has been made in our understanding of the mechanistic changes associated with adopting cryotherapies, research focus tends to look towards the future rather than to the past. Reviewing a topic with a historical perspective, studying a subject considering its earliest phase and subsequent evolution, may help sharpen one’s vision of the present; helping to generate new research questions as well as look at old questions in new ways (Lawrence 1984). Therefore, the aim of this brief historical perspective is to highlight the origins of the many arms of this popular recovery and treatment technique, whilst further assessing the changing face of cryotherapy and discussing what lies ahead in the future for cold-application techniques.

## History of cold-water immersion

The beneficial effects of cold-water immersion (CWI) on human physiology dates as far back as 3500BC with the Edwin Smith Papyrus making numerous references to cold being used for therapeutic purposes (Wang et al. 2006). Ancient Greeks utilised cold-water for therapies as well as relaxation and socialisation; Hippocrates in fourth century BC documenting the use of cold for medicinal purposes and analgesic benefits (Tsoucalas et al. 2015). In Hippocrates’ work entitled “On airs, waters and places”, he makes the statement that “the water can cure everything,” further emphasising its value (Hippocrates, translated by Lloyd, Chadwick & Mann 1950). Cold-water immersion was traditionally used as a treatment for fever with the Roman physician Claudius Galen advocating its use for tertian fever (Wang et al. 2006). The same notion appears hundreds of years later in the work of the physiologist James Currie, who utilised cold-water for treatment of his own fevers (Forrester 2000; Cosby 1950). Currie furthered his interests in the impact of cold-water on human physiology by investigating its influence upon body temperature, pulse, and respiration amongst other parameters. He documented the first records of human temperatures in health, disease, and experimental conditions (Henderson 1971); carrying out such experiments in his own “water cure establishment” to prove the value of hydrotherapy (Tsoucalas et al. 2015).

In the early twentieth century, the physician Edgar A. Hines Jr (1906–1978) advanced our understanding of the physiological responses to cold-water immersion by building upon Bayard T. Horton’s 1927 earlier work describing cold allergy (Lamotte et al. 2021). Hines’ contribution to the scientific and clinical community was to develop the cold-pressor test to study blood pressure variability. In his 1932 seminal paper (Hines and Brown 1932), he showed that immersing the hands in cold-water (4–5 °C for 30 s) led to a different magnitude and time-course of blood pressure response in subjects presenting with hypertension. Hines’ follow-up investigations (Hines and Brown 1933) examining vasomotor reactions to selective sympathectomy being fundamental in interpreting autonomic control of the cold pressor response. Indeed, we now understand that concomitant stimulation of the sympathetic (cold shock) and parasympathetic (diving response; Gooden 1994) arms of the nervous system can lead to autonomic conflict and cardiac arrhythmias (Tipton et al. 2010).

It was not until the 1960s and the work by D H Clarke that cold-water immersion was investigated for benefits to post-exercise recovery (Clarke 1963; Clarke and Stelmach 1966). However, in the decades that followed, attention quickly focussed back towards survival during cold-water exposure; Prof. Mike Tipton (MBE) conducting much work in examining cold shock responses (Tipton 1989), impact of different clothing ensembles (Tipton and Golden 1987; Tipton and Vincent 1989) and adaptations to multiple water immersions (Golden and Tipton 1988). It was at the latter end of the 1990s that the focus of work returned towards determining cold-water immersion’s effects upon performance recovery. Many researchers have since followed the path taken by Paddon-Jones and Quigley (1997) by adopting exercise-induced muscle damaging (eccentric) protocols to track functional, inflammatory and psychophysical responses over several days after cooling. At the turn of the new millennium, a plethora of similar studies followed, employing different exercise modalities, subject cohorts, and/or cooling doses (i.e., duration, water temperature, water depth); the primary aim to inform sports practice. The sheer volume of published studies has led to meta-analyses (Leeder et al. 2012; Poppendieck et al. 2013; Hohenauer et al. 2015; Machado et al. 2016) becoming prevalent in the empirical literature, thus helping form a consensus around the application of cold-water immersion. Current recommendations for the use of CWI derived from these meta-analyses suggest a protocol of 10–15 min at 10–15 °C (Machado et al. 2016) to promote effective recovery, whilst a dose of 1.1 (i.e., 11 min at 10 °C) is required to significantly reduce muscle tissue temperature (Vromans et al. 2019). Elsewhere, it has been established that immediate immersion is preferable versus delayed immersion (Brophy-Williams et al. 2011), whilst the depth of immersion is unlikely to play a significant role (Leeder et al. 2015).

Nevertheless, despite the significant interest, the underpinning physiological mechanisms at the level of the muscle remained largely overlooked. Over the last decade, work shifted towards understanding the central roles that post-immersion changes in muscle temperature (reduction up to − 6.4 °C; Freitage et al. 2021), and limb and cutaneous blood flow (20–30% reduction vascular conductance; Gregson et al. 2011) play in influencing the recovery process. Later studies made use of technological advances to progress earlier work in this area assessing cooling-induced changes in muscle blood flow per se (Ihsan et al. 2013; Mawhinney et al. 2020; Choo et al. 2018). Recent advances in the field of cellular and molecular physiology have also enabled regulatory mechanisms in human skeletal muscle to be studied, developing our insight into important pathways involved in endurance (Joo et al. 2016; Ihsan et al. 2015) and strength (Roberts et al. 2015; Fyfe et al. 2019; Peake et al. 2020) adaptation after cold-water exposure.

## History of ice application

Ice is perhaps recognised as the most traditional mode of cryotherapy; Napoleon’s surgeon to the Grand Army, Baron Dominique Larrey, being one of the earliest proponents in recommending ice and snow to assist in painless amputations and operations on soldiers (Henderson 1971). In the late 1840s, this concept was extended by the physician James Arnott who started using local application of salt solution containing crushed ice to freeze cancerous tumours (Theodorescu 2004); thus, unwittingly developing cryosurgery. However, it was not until the 1960s that the application of ice was recommended for the therapeutic treatment of musculoskeletal injury (Grant 1964; Hayden 1964).

The ability to decrease tissue temperature is fundamental to ice’s therapeutic benefit. In 1955, Bierman studied how ice pack application (120 min) could markedly reduce (~ 6 °C) skin surface temperature (Bierman 1955). Similar studies followed, employing various methods (spray, refrigerant gel, ice pack, ice massage) and cooling durations to subsequently report skin temperature reductions between 6 and 30 °C (see review by Meeusen and Lievens 1986). Indeed, Bleakley and Hopkins (2010) highlighted that crushed ice can reduce skin temperature to below 10 °C after anywhere between 5 and 20 min application. At around the same period as Bierman’s work, Bing and colleagues were one of the first to document the intramuscular temperature change (at 3 cm depth) to ice pack application (Bing et al. 1945). Waylonis (1967) subsequently providing a more comprehensive assessment by recording incremental (0.5 cm) muscle temperature changes to ice massage. However, it should be noted that subcutaneous adipose tissue will influence such temperatures, with Bleakley and Hopkins (2010) highlighting the lowest temperatures seen often coincide with smaller thigh skinfold thickness. It has been suggested that reductions in muscle temperature are related to slowing metabolism, lessening the need for cellular oxygen in the acute period after soft-tissue injury (Swenson et al. 1996). In this regard, much of our current knowledge can be derived back to investigations to conserve organ tissue for transplantation and limb replantation (Sapega et al. 1988; Krezdorn et al. 2017). An early pioneer being the surgeon R. Y. Calne in 1963, who demonstrated that kidneys extracted from Mongrel dogs and cooled with chipped ice could extend the period of ischemia (Calne et al. 1963). Calne’s histochemical analyses showing decreased cellular necrosis within the organ; what we would now term a decrease in secondary ischemic injury (Merrick et al. 1999).

In the 1970s, investigations commenced to better understand the analgesic effect of ice upon the pain threshold (Halliday et al. 1969; Benson and Copp 1974; Bugaj 1975). Bugaj (1975), demonstrating ice massage to acutely abolish the pain threshold (to surface pin prick) when skin temperature was reduced to 13.6 °C. It is now understood that a large part of the analgesic benefit experienced is because of a reduced nerve conduction velocity in sensory nerves (Herrera et al. 2010; Algafly and George 2007; Ernst and Fialka 1994). In 1978, Gabe Mirkin was no doubt influenced by past findings to introduce the widely circulated RICE acronym (rest, ice, compression, and elevation) to guide acute sport injury management. The original acronym subsequently adapted to include RICES (rest, ice, compression, elevation, and stabilisation) (Long and Jutte 2020) and PRICE (protection, rest, ice, compression, and elevation) (Bleakley et al. 2007).

Despite the longstanding popularity of applying ice in sport injury management, the evidence for its use in humans is limited; however, Guillot and colleagues were able to show a positive impact of ice application upon inflammatory cytokines (Guillot et al. 2017, 2019). Nevertheless, recent literature has questioned the use of traditional cold therapy in the early phases of soft-tissue injury (Wang and Ni 2021) or suggests the removal of ice altogether in the management of soft-tissue injuries (Dubois and Esculier 2020). Consequently, the acronyms of PEACE (protection, elevation, avoid anti-inflammatory drugs, compression, and education) and LOVE (load, optimism, vascularization, and exercise) have emerged (Dubois and Esculier 2020). Whilst ice application for acute injury management is still often witnessed pitch side for sporting contact and non-contact injuries, it has more recently been transferred to form part of a periodized recovery approach to facilitate performance (Thorpe 2021).

## History of cold air application

Whilst the use of cold-water and ice cryotherapy methods for exercise recovery have been long established, the application of extreme cold air temperatures (below − 100 °C) is a relatively new technique in the sporting world. The typical use of cold air for recovery is in the form of whole-body cryotherapy (WBC) chambers, which typically exposes an individual to 2–3 min of exposure, after a preceding 30 s temperature adaptation period at approximately 60 °C (Banfi et al. 2010). The cold air, typically administered in the form of either liquid nitrogen or refrigerated cold air (Costello et al. 2016), is proposed to be effective in reducing the sensation of delayed onset of muscle soreness (Hausswirth et al. 2011), increasing parasympathetic activation (Hausswirth et al. 2013) and anti-inflammatory cytokines (Lubkowska et al. 2011; Lombardi et al. 2017).

Initial WBC chambers were built in Japan around 1978, through pioneering work led by Dr. Toshima Yamaguchi for the treatment of rheumatoid arthritis and general pain management. Dr. Yamaguchi’s initial work found that exposure to WBC led to rapid temperature decreases on the outer layers of the person’s skin, resulting in a release of endorphins and subsequent reduction in subjective pain assessment. Indeed, it was reported that around 80% of Dr. Yamaguchi’s patients were completely relieved of their symptoms and chronic pain issues. The success of WBC treatment in Japan led to data being presented at the Rheumatology Congress in 1979; promoting the expansion of its use worldwide. The renowned German rheumatologist, Prof. Reinhard Fricke, bringing WBC treatment to Europe in 1984 and setting up a cryotherapeutic module for hundreds of patients suffering from multiple sclerosis and arthritic conditions.

Despite the popularisation of WBC for the treatment of various conditions, it was not until the past decade that its use crossed over into the sporting world for exercise recovery. Sports such as rugby and American football were early adopters of WBC chambers for recovery, mainly due to the proposed reduction in inflammation that is common in contact sports (Selfe et al. 2014). It is suggested that the extreme temperatures of WBC magnify the effect of cold therapies (such as in comparison to CWI), thus potentially shortening recovery time (Rose et al. 2017). Anecdotally, athletes have reported preferring the use of ‘dry’ therapies such as WBC rather than prolonged immersion in CWI; one reason for its increased popularisation. In the early 2010s, commercial companies were providing sporting organisations with permanent WBC chambers installed within their training base. However, the lack of portability of such chambers limited their practical application. This has led to more recent developments, such as transportable WBC chambers, that can be mounted on a truck for a semi-permanent installation.

Alongside the developments of WBC, a new method called partial-body cryotherapy (PBC) using a portable cryotherapy-cabin has gained popularity within the general public. This system has an open tank and exposes the body, except the head and neck, to similar extreme cold air temperatures (Costello et al. 2016). Whilst PBC has been widely adopted due to its proposed benefits for beauty treatment (e.g., skin care improvements), recreational athletes have started to use such methods for recovery after exercise. PBC cabins have become more widely available in health centres at an affordable cost. Unfortunately, this has coincided with reported cases of severe injury (e.g., skin burns) and death as a result cold air therapy misuse. Moreover, as adverse events must be self-reported, it is likely that the number of events has not been comprehensively documented. Currently, the efficacy of PBC cabins compared to WBC for exercise recovery has yet to be fully determined, with the long-term use of cold air therapy methods (for recovery) still remaining to be clearly established (Malone et al. 2021).

## The changing face of cryotherapy

As the popularity of cryotherapy has increased, clinicians, practitioners, and athletes alike have pursued easy to use, rapid to deploy, and portable cryotherapy alternatives. As an example, development of cryopneumatic devices (e.g., Polar Care, Breg, Inc.; Game Ready, CoolSystems, Inc.; etc.) became popular after receiving patents in the 1990s and early 2000s. Initially implemented for recovery following surgical procedures, such as knee arthroplasty (Su et al. 2012; Murgier et al. 2017; Schinsky et al. 2016), knee arthroscopy (Waterman et al. 2012; Murgier and Cassard 2014), hip arthroplasty (Leegwater et al. 2012), hip arthroscopy (Klaber et al. 2019), and spine surgery (Nabıyev et al. 2018; Bellon et al. 2019), these devices deliver continuous or intermittent compression whilst simultaneously supplying low temperature “exchange fluid” to the injured area. Importantly, they can be applied for as long a time period as desired without substantial change in the temperature of the material contacting the affected area. The result from these studies is highlighting the acute analgesic effect and consistent reduction in patient reported pain. These apparatus presented the advantage of portability and thus were extremely well received by athletes for recovery from exercise. However, to date, only one study has investigated the effect of a cryopneumatic device (as a recovery method) upon physiological and biomechanical outcomes following exercise; showing no benefit in accelerating acute recovery of strength loss (hamstring eccentric strength; Alexander et al. 2021). Ultimately, evidence for the use of cryopneumatic devices for exercise recovery remain anecdotal.

More recently, phase change material (PCM) has been introduced as a modern cryotherapy modality for recovery following exercise (see Kwiecien et al. 2020a for review). A PCM is any substance that goes through a phase transition between states of matter with no detectible temperature change (defined as a latent phase). On the contrary, a substance like water or a gel pack (which does not undergo a change in phase) only experiences a temperature change that can be felt and measured by a thermometer (defined as a sensible phase). A modality with PCM properties is advantageous, because it can absorb large amounts of heat at an almost constant temperature until all the materials are melted; resulting in lower intramuscular temperatures than those which maintain the same phase (Chesterton et al. 2002; Merrick et al. 2003; Vieira et al. 2013). Compared to the conventional cryotherapy interventions, PCM offers an advantage over traditional cryotherapy modalities in that it can be safely administered for prolonged durations (Kwiecien et al. 2019), resulting in a larger magnitude of change in tissue temperature (Merrick et al. 2003; Dykstra et al. 2009).

In particular, PCM packs with a latent phase of 15 °C (Glacier Tek USDA BioPreferred PureTemp PCM, Plymouth, MN, USA) can be safely administered for durations of 3 (Brownstein et al. 2019; Clifford et al. 2018; Kwiecien et al. 2019, 2021; Mullaney et al. 2021) to 6 (Kwiecien et al. 2018; Kwiecien et al. 2020b) h at a time in one dose. Indeed, 6 h of 15 °C PCM cooling has been shown to accelerate recovery of strength loss and soreness following isolated eccentric exercise of the quadriceps muscle in untrained (Kwiecien et al. 2018) and trained (Kwiecien et al. 2020b) individuals. Similarly, 3 h of PCM cooling has also been shown to accelerate recovery of strength loss (Brownstein et al. 2019; Clifford et al. 2018) and soreness (Clifford et al. 2018) following soccer match play and baseball pitching (Mullaney et al. 2021) but not following a marathon run (Kwiecien et al. 2021). Nevertheless, the efficacy of prolonged PCM cooling for accelerating recovery from “injury” remains to be investigated. It should be noted that anecdotal application of these evolving techniques likely includes multiple and combined use. However, as research surrounding these techniques is emerging, the interchangeability of these cryotherapy alternatives is currently unknown.

## The future of cryotherapy

Whilst the method of application is open to change, the mechanisms of action remain the same. Whether the cooling stimulus applied is water, ice, air, or PCM, future research should direct attention towards ensuring that appropriate protocols are utilised. In this sense, an appropriate protocol should be able to elicit the necessary physiological alterations proposed to benefit health, injury, or recovery (Kwiecien and McHugh 2021). This is undoubtedly likely to change depending on the method of cryotherapy used. Indeed, an important point to note is the impact of the thermal gradient created between the skin and the surrounding environment (Bleakley et al. 2014). The thermal conductivity, or heat-transfer co-efficient, is much greater for ice (2.18 k), when compared with water (0.58 k) and air (0.024 k), suggesting a greater ability for the removal of heat from the body using ice. However, despite these values, water and air may be more efficient through greater surface area contact (Bleakley et al. 2014). Therefore, the thermal properties and rate of heat exchange, temperature and duration of cooling, and size of area exposed to cooling should all be carefully considered. With data existing directly comparing the efficacy of cryotherapy modalities on physiological parameters (Abaïdia et al. 2017; Costello et al. 2012; Wilson et al. 2018; Mawhinney et al. 2017b), it is important that practitioners are aware that some methods may be more efficient in the removal of heat than others.

One thing that remains important is the correct and efficient transfer of knowledge between scientific and applied communities (Allan et al. 2021). When applying scientific data to practice, it is important that the correct context is utilised (Ihsan et al. 2021). Recent expert views suggest cryotherapies that aim to benefit or improve health, injury, or recovery should be implemented in an individualised and periodised manner that takes into account the athlete, training and competition schedule, session aims, proximity of future exercise, and environmental conditions (Ihsan et al. 2021; Ihsan et al. 2020; Allan and Mawhinney 2017, Grainger et al. 2021). Whilst readers are directed to recent useful reviews highlighting the positive effects of cryotherapies upon health, injury, and recovery (Kwiecien and McHugh 2021), they should also be aware of important caveats that might arise in specific situations; for example, the paradox whereby post-exercise cooling might enhance endurance-based adaptations in skeletal muscle (Ihsan et al. 2014, 2015, 2020; Aguiar et al. 2016; Joo et al. 2016; Allan et al. 2017, 2019, 2020; Broatch et al. 2017) but dampen hypertrophic aims (Roberts et al. 2015; Fuchs et al. 2020). A point we have previously discussed (Ihsan et al. 2021) and one that serves to emphasise the need for individualisation and periodisation of recovery strategies.

What does remain clear is the firm centuries old belief in cryotherapy techniques and their analgesic properties. Recent data, focussing on the perception of one post-exercise cooling strategy (CWI), highlight the positive perception surrounding its application, with “end-user belief” being an important variable considered before use (Allan et al. 2021). Whilst we now have the capabilities to examine these age-old theories, researchers should look to further establish the importance of cooling on health, wellness, and well-being.

## Data Availability

Not applicable.

## Abbreviations

CWI: Cold-water immersion
PBC: Partial-body cryotherapy
PCM: Phase change material
WBC: Whole-body cryotherapy
